# A novel adenovirus isolated from the Egyptian fruit bat in South Africa is closely related to recent isolates from China

**DOI:** 10.1038/s41598-018-27836-w

**Published:** 2018-06-25

**Authors:** Petrus Jansen van Vuren, Mushal Allam, Michael R. Wiley, Arshad Ismail, Nadia Storm, Monica Birkhead, Wanda Markotter, Gustavo Palacios, Janusz T. Paweska

**Affiliations:** 1 Centre for Emerging Zoonotic and Parasitic Diseases, National Institute for Communicable Diseases of the National Health Laboratory Service, Sandringham, Johannesburg South Africa; 2 Centre for Viral Zoonoses, Department of Medical Virology, Faculty of Health Sciences, University of Pretoria, Pretoria, South Africa; 3 Core Sequencing Facility, National Institute for Communicable Diseases of the National Health Laboratory Service, Sandringham, Johannesburg South Africa; 4 Center for Genome Sciences, United States Army Medical Research Institute of Infectious Diseases, Fort Detrick, USA; 5 Faculty of Health Sciences, University of the Witwatersrand, Johannesburg, South Africa

## Abstract

Recently a number of novel adenoviruses have been isolated from diverse bat species and from diverse geographical locations. We describe the isolation of a novel adenovirus (Family *Adenoviridae*, genus *Mastadenovirus*) from a pool of liver and spleen tissue of an apparently healthy wild-caught Egyptian fruit bat (*Rousettus aegyptiacus*) in South Africa. Genetically the virus is most closely related to four mastadenoviruses recently isolated in China, from *Miniopterus schreibersi* and *Rousettus leschenaultii* bats, which are highly divergent from previously identified bat adenoviruses. The length of the *Rousettus aegyptiacus* adenovirus-3085 (RaegAdV-3085) genome, at 29,342 bp is similar to its closest relatives, and contains 27 open reading frames. The RaegAdV-3085 genome has a low G + C content (36.4%) relative to other viruses in the genus (between 43.6 and 63.9%) but similar to its closest relatives. The inverted terminal repeat (ITR) of RaegAdV-3085 is only 40 bp compared to between 61 and 178 bp of its closest relatives. The discovery of RaegAdV-3085 expands the diversity of known adenoviruses in bats and might represent a member of a new mastadenovirus species in bats.

## Introduction

Adenoviruses (family *Adenoviridae*) are non-enveloped double strand DNA viruses, 70–90 nm in diameter and containing a single linear genome ranging in size from 26 to 48 kb. The genome contains an inverted terminal repetition (ITR) which ranges in size from 36 to 371 bp between different viruses. There are currently five recognized genera: *Atadenovirus, Aviadenovirus, Ichtadenovirus, Mastadenovirus* and *Siadenovirus*^1^. Adenoviruses infect a wide range of hosts. The *Mastadenovirus* genus is the largest, containing 22 recognized species, isolated exclusively from mammals including human, bovine, bat, canine and equine hosts. Other adenoviruses from mammalian hosts are from species within the *Atadenovirus* genus which also includes viruses from avian and serpentine hosts. The *Aviadenovirus* genus comprises exclusively avian viruses; the *Ichtadenovirus* genus contains one species isolated from sturgeon fish, while *Siadenovirus* genus includes viruses isolated from frogs and birds. The *Mastadenovirus* genus includes a number of unclassified viruses isolated from bats^2–9^. It has been recently proposed and accepted to classify a number of these viruses into two new species, *Bat mastadenovirus A* and *B*^10^.

Adenoviruses have been isolated from bats from various geographical locations and different bat species. The first adenovirus from a bat was isolated in Japan from a fruit-eating flying fox in 2006 (*Pteropus dasymallus yayeyamae*)^9^. In China, adenoviruses have been isolated from the insectivorous common bent-wing (*Miniopterus schreibersii*) and Rickett’s big-footed bats (*Myotis ricketti*), as well as the fruit-eating Leschnault’s rousette bat (*Rousettus leschenaulti*)^5–7^. In Germany these viruses have been isolated from the insectivorous common pipistrelle bat (*Pipistrellus pipistrellus*)^3,4^. In the USA a novel adenovirus was isolated from a Rafinesque’s big-eared bat (*Corynorhinus rafinesquii*)^2^. Metagenomic or PCR-based detection studies have revealed the presence of adenoviruses in the guano of greater mouse-eared bat (*Myotis myoits*) in Germany^11^, common noctule (*Nyctalus noctula*) and greater horseshoe bats (*Rhinolophus ferrumequinum*) in Hungary^12^, and the pallid bat (*Antrozous pallidus*) in the USA^13^. One bat associated adenovirus has been isolated from the straw coloured fruit bat (*Eidolon helvum*) in Ghana, Africa^8^.

Adenovirus infections usually result in mild disease in different hosts with a few exceptions. In dogs, canine adenovirus 1 (CAdV-1) and CAdV-2 cause infectious hepatitis and respiratory disease respectively^14,15^. Human diseases caused by adenoviruses include respiratory illness, conjunctivitis, hepatitis and gastroenteritis^6^. Fatal human pneumonia has also been noted due to adenovirus infection^16^. A severe adenovirus outbreak in a colony of captive monkeys in a Californian research facility, followed by primary transmission to one of the researchers and secondary transmission to one of his family members highlights the potential of zoonotic spillover of non-human adenoviruses and consequent human-to-human transmission^17^.

Here we describe the discovery, genomic and electron-microscopic characterization of a novel mastadenovirus, named *Rousettus aegyptiacus* adenovirus-3085 (RaegAdV-3085), isolated from a pool of liver and spleen tissues of an apparently healthy Egyptian fruit bat (*Rousettus aegyptiacus*) in South Africa.

## Results

### Virus isolation

**Figure 1:**
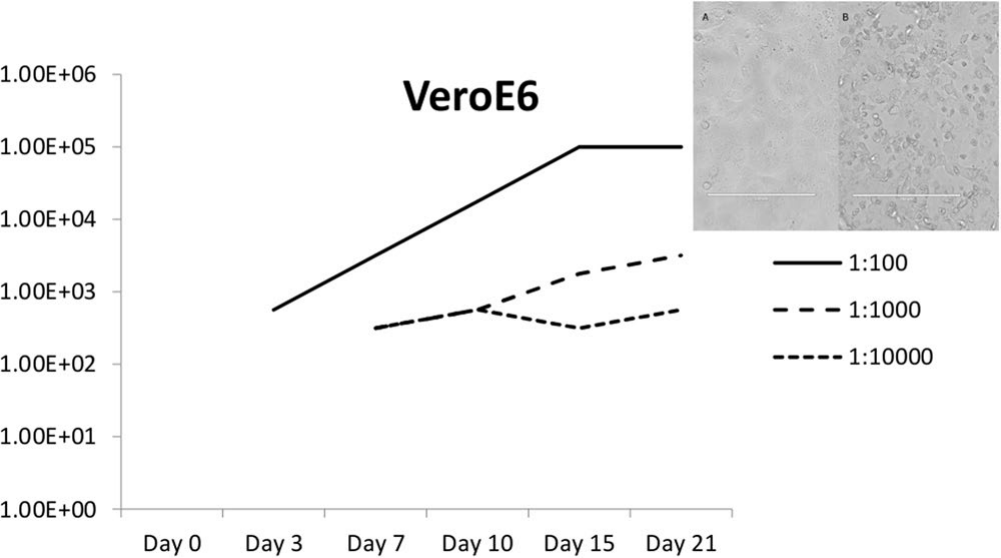
RaegAdV-3085 growth curve in VeroE6 cell culture. Different line types indicate different dilutions of stock virus. Y-axis represents the TCID_50_/mL of the virus in culture supernatant. The inset shows uninfected (A) and infected (B) VeroE6 cells with CPE at 12 days post inoculation.

A virus, causing cytopathic effect (CPE) was isolated from the liver/spleen homogenate of bat 3085, after three passages in Vero cells. The Egyptian fruit bat from which the virus was isolated, was collected in December 2013 as part of routine surveillance for bat borne viruses in South African bat populations. The virus infection resulted in disruption of the Vero cell monolayer by day 12 post infection, causing infected cells to shrink, become round and more refractory to light, and finally detach from the tissue culture plastic surface (inset A and B, Fig. 1). The virus replicates relatively slow in VeroE6 cells, reaching a peak in virus titre 15 days after inoculation (Fig. 1). The virus did not replicate in HEK293 cells (results not shown).

### Identification and characterization by electron microscopy

**Figure 2:**
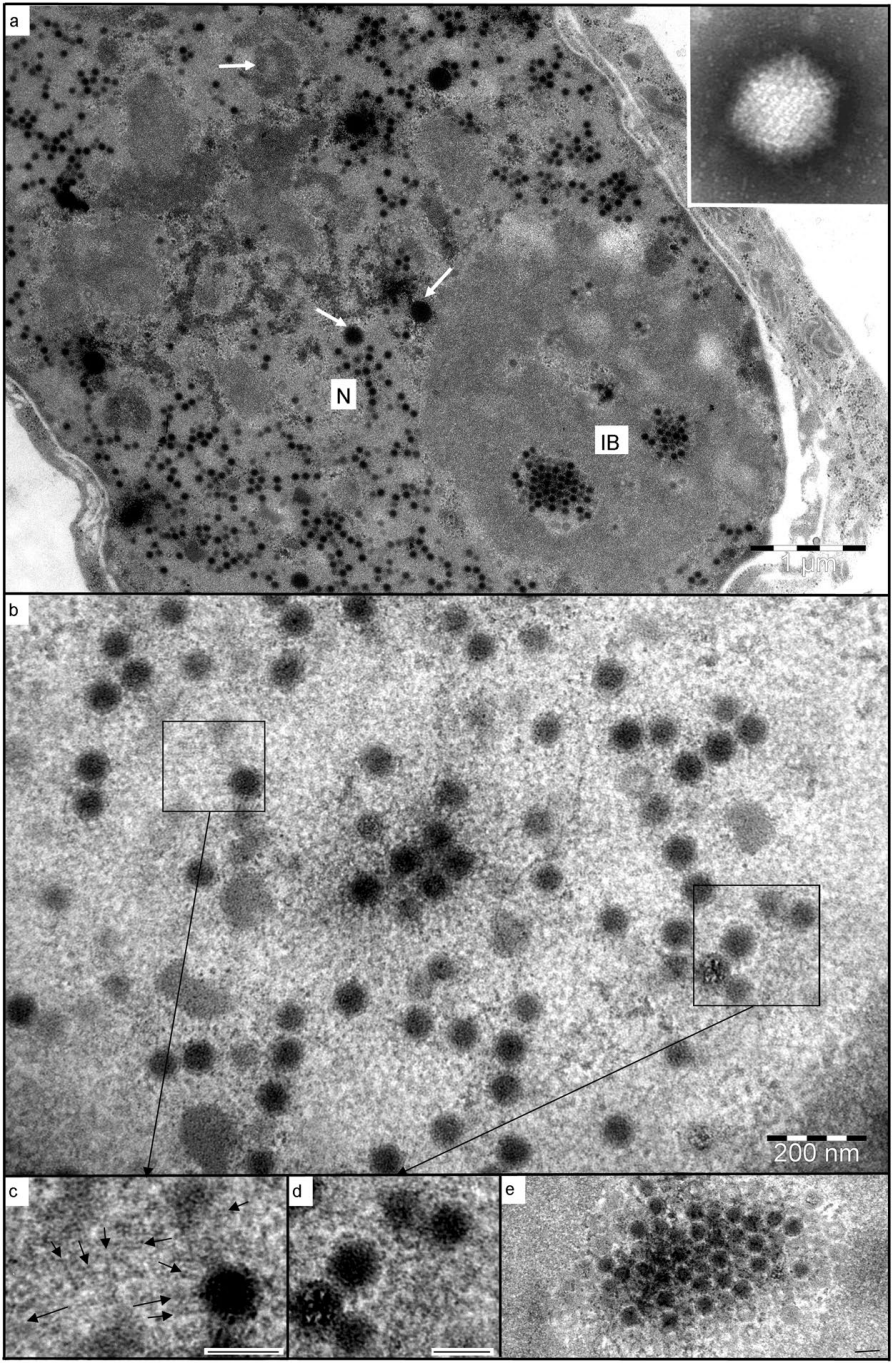
Electron microscopy of RaegAdV-3085 infected Vero cells. (**a**) Numerous virions forming within the nucleus (N), with crystalline arrays evident within the granular matrix of an inclusion body (IB). Remnants of probable nuclear bodies are scattered throughout the nucleus (arrows). INSET: a negatively-stained virion; (**b**) developing virus particles within an inclusion body; (**c**) actin-like filaments (arrows) associated with developing virion; (**d**) fine, proteinaceous filaments extending between developing virions; (**e**) one of a series of serial sections through a crystalline array, showing the predominance of empty capsids around the periphery. Scale bars (**d**,**e**,**f**) = 80 nm.

Negatively-stained virions were identifiable as adenovirus, being 70 nm in diameter, non-enveloped, and with spherical capsomers forming distinct facets of the icosahedra (Fig. 2a inset). Resin sections through infected Vero cells confirmed this finding, with numerous virus particles being formed within the nuclei (Fig. 2a), prior to lytic release from the cytoplasm. As the cells were in the late phase of infection, disruption and dispersal of the promyelocytic leukaemia bodies and Cajal bodies had occurred^18^, with only smaller, scattered remnants of these nuclear bodies evident (Fig. 2a).The similarities between adenovirus replication centres and nucleoli^19^ were evident in infected nuclei, with the underlying granular component forming a matrix in which virus particles developed in association with fibrillar components of differing osmiophilic densities (Fig. 2b–d). Nuclear actin filaments were seen in association with newly-forming capsids (Fig. 2b,c), similar to those described for alpha-herpesvirus neuronal infections^20^. Delicate, branched filaments were also visible around developing virus particles (Fig. 2d), consistent with the description and occurrence of viral E4-ORF3 protein^21^. Developing virus particles were typically filled with viral genomic material resembling host heterochromatin^22^, but empty capsids were frequently seen around the peripheries of the virus factories (Fig. 2e) – as in both Polyomavirus-infected cells^23^ and a novel strain of porcine adenovirus^24^.

### Preliminary identification by non-biased next generation sequencing

Initial attempts to identify the virus, concurrently with electron microscopy, were done by sequence independent single primer amplification (SISPA) as described recently for two novel RNA viruses we identified^25,26^. NGS following this approach yielded 14 contigs matching adenovirus sequences available on Genbank by dc-megablast. The number of reads making up the 14 contigs ranged from two to 1132 and contig lengths from 103 to 4405 nucleotides (Table 1).

**Table 1:** Contigs from Illumina sequencing of RaegAdV-3085, Vero passage 3, using SISPA approach.

Contig ID	Matching sequence accession number	Matching sequence description on Genbank	Matching sequence start position	Matching sequence end position	Number of reads in contig	Contig length	Percentage sequence identity
c3	KF633445.1	Human_adenovirus_B_/DEU/HEIM_00086	20889	20999	14	161	72.97
c16	AB686663.1	Human_adenovirus_31_R187	162	62	3	104	75.25
c24	DQ630756.1	Ovine_adenovirus_3_PX611	1827	1982	5	157	80.38
c25	AF153447.1	Ovine_adenovirus_A	355	428	7	127	82.43
c43	DQ630755.1	Ovine_adenovirus_2_PX515	1847	1720	6	171	85.94
c49	KC692426.1	Unidentified_adenovirus_PgAdV-10	185	278	2	107	80.85
c64	AF258784.1	Tree_shrew_adenovirus_1	18638	18537	11	103	74.51
c67	HQ241818.1	Simian_adenovirus_48_AJ75	23547	23621	15	230	82.67
c70	HQ605912.1	Simian_adenovirus_20_VR-541	23847	23728	9	184	74.17
c71	Y07760.1	Canine_adenovirus_type_1	22885	22763	7	156	73.98
c75	U40839.3	Ovine_adenovirus_7	12098	11983	2	129	74.14
c82	KF268310.1	Human_adenovirus_C/USA/Pitts_00109/1992/2	26840	26756	7	107	75.29
c66	JX885602.1	Eidolon_helvum_adenovirus_1	832	1662	1132	4405	73.54
c78	JX885602.1	Eidolon_helvum_adenovirus_1	2124	1948	16	186	77.97

### Complete genome sequencing, annotation and phylogenetic analysis

The approach of direct Nextera DNA library preparation for MiSeq sequencing from DNA extracted from concentrated adenovirus (ultracentrifuged cell culture virus particles) yielded 716 contigs after *de novo* assembly, including one contig of 29,301 nucleotides. The 716 contigs were obtained from 912,422 cleaned paired reads, of which the 29,301 nt contig consisted of 232,419 paired reads (25% of total clean reads). The terminal ends of the genome, including a 40 bp ITR, were better resolved after bowtie buildout yielding a sequence of 29,342 nucleotides, encompassing likely the entire genome of RaegAdV-3085 (MG551742). BLAST analysis of this genome revealed highest similarity to Bat mastadenovirus WIV13 complete genome (KT698852)^7^ and *Eidolon helvum* adenovirus 1 hexon gene (JX88562)^8^. The RaegAdV-3085 genome shares a nucleotide identity of 70–74% with complete genomes of Bat mastadenoviruses WIV-12, 13, 17 and −18 (KT698856, KT698852, KX961095, KX961096), and 74% with a partial genome sequence (Hexon gene) of *Eidolon helvum* adenovirus 1 hexon gene (JX88562).

**Figure 3:**
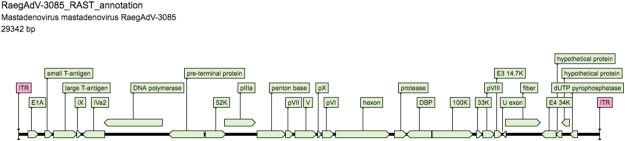
Genomic organization of RaegAdV-3085 genome (29,342 bp). Open reading frames and predicted gene names are shown.

The length of the RaegAdV-3085 genome is smaller than that of most other mastadenoviruses, specifically bat associated adenoviruses, but similar to its closest related viruses from China^7^. The known ITR length range for most mastadenoviruses is 93 to 371 nt, with lengths of between 50 and 73 noted in bat mastadenoviruses, suggesting that the terminal ends of RaegAdV-3085 might not have been fully resolved^2^. The RaegAdV-3085 genome has a G + C content of 36.4%, significantly lower than other recognized mastadenoviruses (43.6 to 63.9%)^1^, but similar to recently isolated bat adenoviruses from China^7^. The RaegAdV-3085 genome contains 27 open reading frames (ORFs) with recognizable similarity to known adenovirus proteins (Table 2; Fig. 3).

**Table 2:** Open reading frames and ITRs in the 29,342 bp genome of RaegAdV-3085.

ORF number	Gene/protein designation or feature	Predicted description or function of protein	Structural or non-structural	Gene (nt)	Protein (aa)	Genomic position
	ITR	Forms panhandles for replication	N/A	40	N/A	1–40
1	E1A	Transccriptional activator	Non-structural	516	171	456–971
2	E1B small	Small T-antigen	Non-structural	372	123	1,324–1,695
3	E1B large	Large T-antigen	Non-structural	1,173	390	1,755–2,927
4	IX	Minor capsid protein	Structural	249	82	2,942–3,190
5	IVa2	DNA packaging ATPase	Structural	1,110	369	3,214–4,323 (c-strand)
6	pol	DNA polymerase	Non-structural	2,922	973	4,311–7,232 (c-strand)
7	pTP	Terminal protein	Structural	1,794	597	7,568–9,361 (c-strand)
8	52 K	DNA packaging protein	Non-structural	1,014	337	9,415–10,428
9	pIIIa	Minor capsid protein	Structural	1,566	521	10,382–11,947
10	Penton	Major capsid protein	Structural	1,425	474	12,029–13,453
11	pVII	Major core protein	Structural	390	129	13,459–13,848
12	V	Minor core protein	Structural	1,101	366	13,929–15,029
13	pX (µ)	Minor core protein	Structural	198	65	15,084–15,281
14	pVI	Minor capsid protein	Structural	600	199	15,324–15,923
15	Hexon	Major capsid protein	Structural	2,727	908	15,988–18,714
16	AVP	Protease	Structural	603	200	18,943–19,545
17	DBP	DNA binding protein	Non-structural	1,260	419	19,583–20,842 (c-strand)
18	100 K	Hexon scaffold protein	Non-structural	2,049	682	20,853–22,901
19	33 K	DNA packaging/assembly protein	Non-structural	291	96	23,087–23,377
20	pVIII	Minor capsid protein	Structural	585	194	23,428–24,012
21	E3 14.7 K	Immuno-modulatory protein	Non-structural	372	123	24,016–24,387
22	U exon	Replication centre protein	Non-structural	168	55	24,399–24,566 (c-strand)
23	Fiber protein	Major capsid protein	Structural	1,773	590	24,565–26,337
24	E4 ORF34K	p53 and p73 inhibitor	Non-structural	738	245	26,368–27,105 (c-strand)
25	Hypothetical protein	Unknown	Unknown	309	102	27,118–27,426 (c-strand)
26	Hypothetical protein	Unknown	Unknown	396	131	27,420–27,815 (c-strand)
27	dUTP pyrophosphatase	pyrophosphatase	Non-structural	393	130	27,809–28,201 (c-strand)
	ITR	Form panhandles for replication	N/A	40	N/A	29,303–29,342

The putative penton protein of RaegAdV-3085 contains an Arg-Gly-Asp (RGD) motif at position 271–271 (amino acid sequence), which is a common feature of many human adenoviruses involved in binding cell surface integrins^27^. Similar to BtAdV-WIV12, 13, 17 and 18, RaegAdV-3085 encodes only one putative E3 protein of 14.7 K.

**Figure 4:**
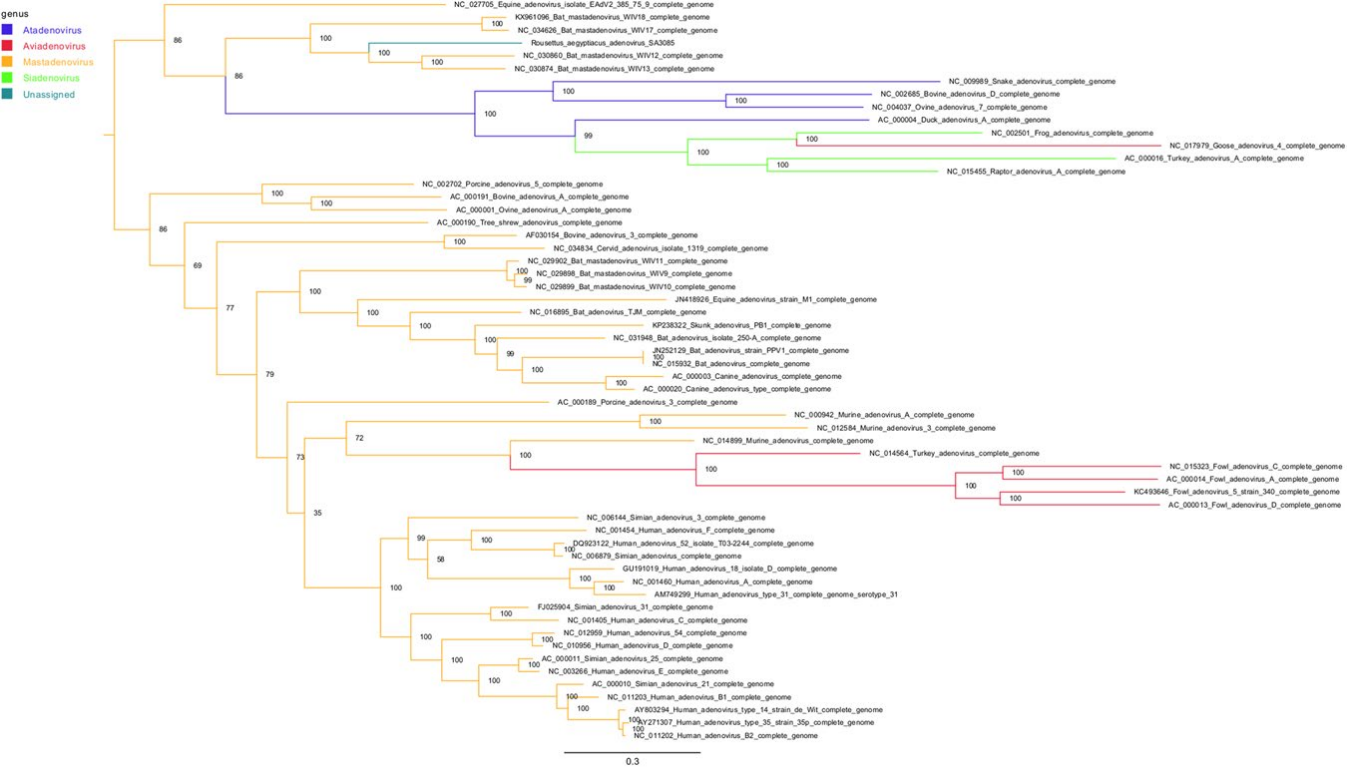
Maximum likelihood analysis (RAxML) of the RaegAdV-3085 genome with genomes of representative viruses from all genera within the *Adenoviridae* family. Branches are coloured according to the genus to which the respective viruses belong, as indicated in the legend. The tree is rooted at the midpoint and nodes ordered in decreasing order.

**Figure 5:**
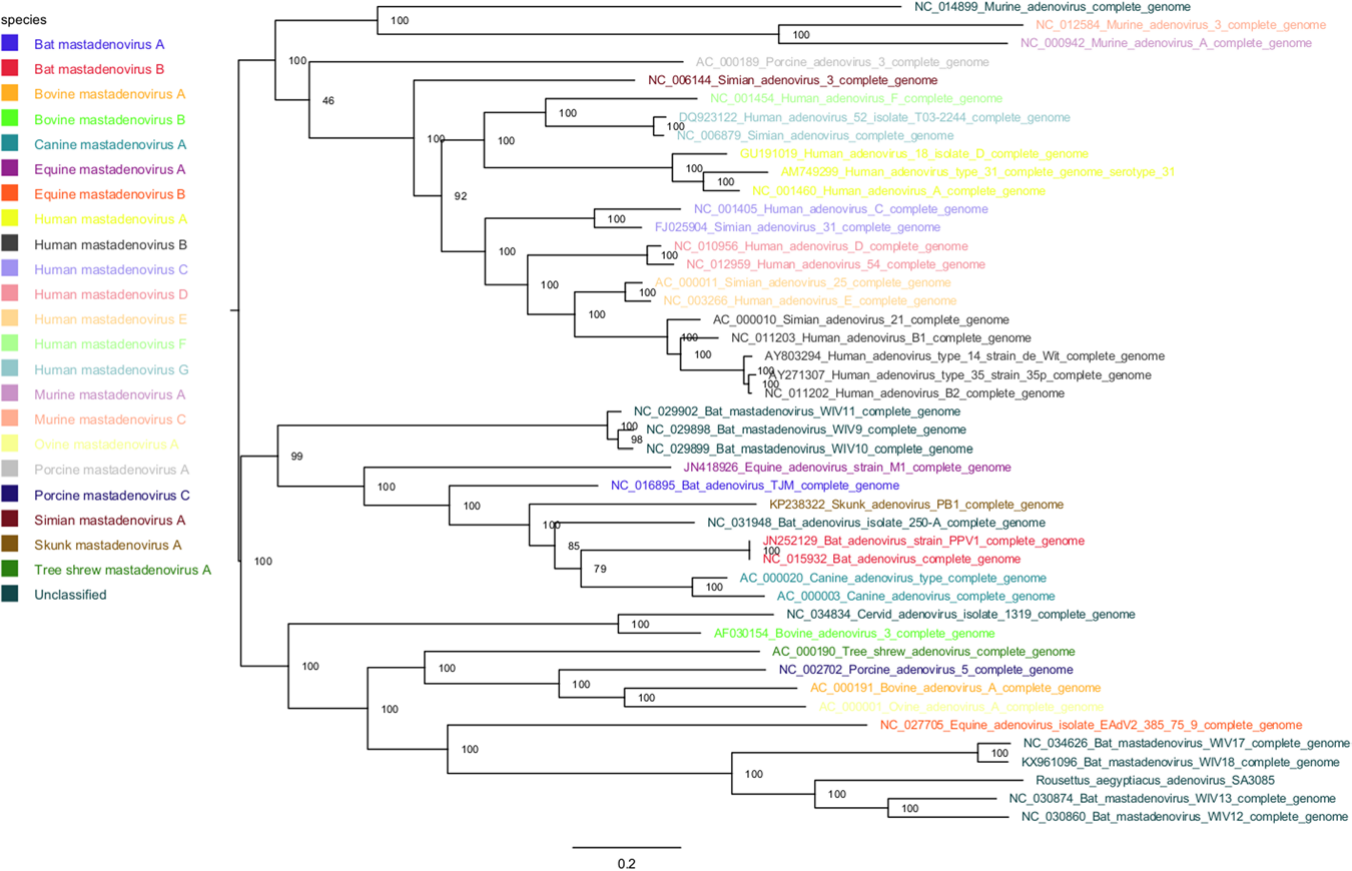
Maximum likelihood analysis (RAxML) of the RaegAdV-3085 genome with genomes of representative viruses from different species within the *Mastadenovirus* genus. Sequence names are coloured according to the species to which they belong (legend). The tree is rooted at the midpoint and nodes ordered in decreasing order.

Phylogenetic analysis confirms that the RaegAdV-3085 virus belongs to the *Mastadenovirus* genus in the *Adenoviridae* family (Fig. 4). Within the genus, the virus forms a clade with the four viruses isolated from bats in China^7^. Within the clade, RaegAdV-3085 seems to group more closely with WIV-12 and WIV-13, both of which were isolated from *Miniopterus schreibersii* bats, as opposed to WIV-17 and WIV-18 that were both isolated from *Rousettus leschenaulti* bats (Fig. 5).

## Discussion

We describe the isolation, identification, electron-microscopic and sequence characterization of a novel adenovirus isolated from an Egyptian fruit bat in South Africa. The source animal was apparently healthy and was sampled as part of surveillance for bat-borne zoonotic pathogens, in a cave situated in Limpopo Province. The virus, provisionally named *Rousettus aegyptiacus* adenovirus-3085, abbreviated to RaegAdV-3085, groups phylogenetically with the *Mastadenovirus* genus and shares genome organization characteristics.

Bat-associated adenoviruses are diverse in their DNA sequence as well as genome size and G + C content^6,7^. The new virus described here further demonstrates this since it is only 74% related to its closest relative based on full genome DNA sequence, and has a relatively short genome with low G + C content. Based on phylogenetic analysis the virus falls within a clade formed by four viruses isolated recently in China. Interestingly, two of these viruses were isolated from the *Rousettus leschenaulti* fruit bat, a species related to the Egyptian fruit bat, yet RaegAdV-3085 is more closely related to the other two viruses isolated from insectivorous common bent-wing bats (*Miniopterus schreibersii*). This is surprising considering that adenoviruses are thought to have co-evolved with their hosts^7^. This observation might be explained by the fact that relatively few bat-associated adenoviruses have been isolated and characterized to date, thus the phylogeny might only be resolved better once more representative adenoviruses from more bat species have been sequenced. In fact, the first adenovirus from bats was only discovered ten years ago^9^. Another possible explanation might reside in the knowledge of the ecological niche of the particular Egyptian fruit bat population studied here. The cave hosting these bats is co-inhabited by up to three different insectivorous bat species, including a large seasonal colony of the Natal long-fingered bat (*Miniopterus natalensis*), a close relative of the common bent-wing bat. It is therefore not irrational to hypothesize that the Egyptian fruit bat from which the virus was isolated might have been only coincidently infected through close contact with co-roosting Natal long-fingered bats. This can, however, only be concluded when more sequences become available from these potential hosts. Both of these bat species are widely distributed with overlapping ranges; the Natal long fingered bat is distributed across Southern- and East Africa, into the Arabian Peninsula, while the Egyptian fruit bat is additionally found in West Africa, North Africa and south-west Asia.

The reservoir status will have to be addressed in future studies aimed at surveillance for RaegAdV-3085 infected bats in the cave (and other) through molecular and/or serological testing. Also useful would be experimental infection studies of suspected host reservoir species to evaluate pathological, virological and serological responses. Such studies might also yield data on transmission routes between bats and possible maintenance mechanisms. A wide number of viruses have been detected in or isolated from bats in the last decade due to an exponential increase in the interest in bats and their role in harbouring dangerous zoonotic pathogens. The transmission routes of a number of these viruses between bats, or from bats to other species, are known or can be inferred from the tissues in which they were detected. However, there are still viruses, for which bats have been determined or are hypothesized to be reservoir hosts, such as the filoviruses where the exact transmission routes remain enigmatic^28^. Elucidating such mechanisms for more bat-associated viruses might shed more light in general on how bats can maintain and transmit pathogens of human and veterinary health importance.

The fact that RaegAdV-3085 contains the Arg-Gly-Asp (RGD) motif in its putative penton protein sequence, usually a common feature of human adenoviruses that plays a role in binding cell surface integrins, and the ability of the virus to replicate in non-human primate cells *in vitro* (Vero), suggests that the virus might not be restricted only to replication in bats. Although adenoviruses are relatively host-specific, it was shown recently that another bat adenovirus (BtAdV 250-A) replicates efficiently in a range of host cells *in vitro*, from fox to monkey cells^2^. Thus it seems like bat associated adenoviruses in general are able to infect a wider range of host cells, including human cells *in vitro*^5^.

An interesting aspect to consider in research into a better understanding of maintenance and transmission of dangerous zoonotic pathogens in bat populations is the possibility of co-infections and how these affect the immune status of bats, subsequent shedding patterns, and even recrudescence of possible latent infections due to an overburdened immune system. With the high number of pathogens recently identified in bats, it is possible to imagine the likelihood of a bat being co-infected or infected with different pathogens in short succession, leading to a weakened immune system and possibly increased shedding.

The isolation of RaegAdV-3085 expands the repertoire of adenoviruses isolated from bats as well as their geographical distribution. Additionally, it highlights the likely wealth of undiscovered bat associated (but also other mammalian) adenoviruses and the need to intensify efforts to better resolve our knowledge on the evolution of these viruses as well as cross-species transmission events.

## Methods

### Virus isolation and source animals

Egyptian fruit bats (*Rousettus aegyptiacus*) were sampled between March 2013 and March 2014 at Matlapitsi cave in the Matlapitsi Valley, Limpopo Province, South Africa, as described before^25,26^. Tissues were collected from a total of 102 of these bats. Liver and spleen pools (combined ± 0.1 g) were homogenized (30 Hz for 8 minutes using a Tissuelyzer II and 5 mm stainless steel beads, Qiagen) in Eagle’s minimum essential medium (10% w/v) and clarified supernatants used to inoculate Vero cell cultures, and monitored as described before^25,26^. A growth curve experiment in VeroE6 cells was performed as follows. Stock RaegAdV-3085 (1 × 10^5.7^ TCID_50_/mL) was diluted 10^−2^; 10^−3^ and 10^−4^ in culture medium, and 5 mL of each dilution used to inoculate 75 cm^2^ culture flasks containing 95% confluent VeroE6 monolayers. The inoculum was removed after 1 hour and fresh culture medium added, after which the flasks were incubated at 37 °C in 5% CO_2_ for 21 days. Aliquots of culture supernatant was collected on the day of inoculation, and thereafter on days 3, 7, 10, 15 and 21 from all three flasks. Supernatant collections were subjected to standard TCID_50_ titration as described previously^26^.

### Sequencing

Initially, sequencing was performed as described previously using a sequence-independent single primer amplification (SISPA) method, biased towards RNA amplification^25^. When it became apparent that the unknown virus was a DNA virus, an alternative method was followed. Virus was propagated and concentrated as described previously with some modification^29^. Infected cells were harvested at 75% CPE, freeze-thawed three times and centrifuged at 3000 × *g* at 4 °C for 15 minutes to remove cell debris. The virus was pelleted by ultracentrifugation and resuspended in TE buffer (pH 8.0). A volume of 20 ml virus suspension was added to the 30 ml centrifuge tube, and 10 ml ice-cold sucrose solution (30% w/w) layered beneath it. The tube was centrifuged at 141 000 × *g* in a Beckman SW-28 rotor for 90 minutes at 4 °C. The supernatant was removed and the pellet resuspended in 0.5 ml of TE buffer. Total DNA was then extracted from the concentrated virus stock using a Qiagen DNA blood mini-kit. Libraries were prepared using the Nextera DNA library preparation kit and sequencing performed on an Illumina MiSeq instrument.

### Transmission electron microscopy

Processing of infected cell cultures for transmission electron microscopy was performed as described previously^25,26^. Briefly, culture supernatant was concentrated, adsorbed onto Formvar-coated grids, negatively stained with saturated, aqueous uranyl acetate and viewed. Infected monolayers were routinely processed for ultramicrotomy (primary fixation in 2.5% glutaraldehyde in 0.1 M sodium cacodylate buffer pH 6.9, post-fixation in 1% osmium tetroxide, embedding in a low-viscosity resin, double-staining of 70 nm sections).

### Phylogenetic and sequence analysis

Illumina sequence data was processed as described previously^25^: quality filtering was conducted with Prinseq-lite and reads assembled into contigs using Ray Meta with kmer length = 25. The complete sequence of RaegAdV-3085 was obtained in one contig (29,342 bp). Alignment with sequences available on the NCBI-Nucleotide database (Genbank) was performed using MAFFT v7.222^30^. Maximum likelihood analysis was performed using RAxML v8.2^31^. The genome was annotated using Prokka^32^ and WebDSV (http://www.molbiotools.com/WebDSV/).

### Ethical Statement and regulatory requirements

This study was carried out according to the recommendations of the South African National Standards for the Care and Use of Animals for Scientific Purposes (SANS 10386: 2008). The field sampling protocols and transport of *Rousettus aegyptiacus* and samples collected from this species are approved by the National Health Laboratory Service Animal Ethics Committee (AEC 137/12), University of Pretoria Animal Ethics Committee (EC054–14), Department of Economic Development, Environment and Tourism: Limpopo Province Directorate: Wildlife Trade and Regulation Permit (CPM 006806) and the South African Department of Agriculture, Forestry and Fisheries (Section 20 approval 12/11/1/1/8).

### Genbank accession numbers

MG551742.
